# Comparison of Anterior Segment Measurements with Scheimpflug/Placido Photography-Based Topography System and IOLMaster Partial Coherence Interferometry in Patients with Cataracts

**DOI:** 10.1155/2014/540760

**Published:** 2014-10-23

**Authors:** Jinhai Huang, Na Liao, Giacomo Savini, Fangjun Bao, Ye Yu, Weicong Lu, Qingjie Hu, Qinmei Wang

**Affiliations:** ^1^School of Optometry and Ophthalmology and Eye Hospital, Wenzhou Medical University, 270 West Xueyuan Road, Wenzhou, Zhejiang 325027, China; ^2^Key Laboratory of Vision Science, Ministry of Health, 270 West Xueyuan Road, Wenzhou, Zhejiang 325027, China; ^3^Studio Oculistico d'Azeglio, Bologna, Italy

## Abstract

*Purpose*. To assess the consistency of anterior segment measurements obtained using a Sirius Scheimpflug/Placido photography-based topography system (CSO, Italy) and IOLMaster partial coherence interferometry (Carl Zeiss Meditec, Germany) in eyes with cataracts.* Methods*. A total of 90 eyes of 90 patients were included in this prospective study. The anterior chamber depth (ACD), keratometry (K), corneal astigmatism axis, and white to white (WTW) values were randomly measured three times with Sirius and IOLMaster. Concordance between them was assessed by calculating 95% limits of agreement (LoA).* Results*. The ACD and K taken with the Sirius were statistically significantly higher than that taken with the IOLMaster; however, the Sirius significantly underestimated the WTW values compared with the IOLMaster. Good agreement was found for Km and ACD measurements, with 95% LoA of −0.20 to 0.54 mm and −0.16 to 0.34 mm, respectively. Poor agreement was observed for astigmatism axis and WTW measurements, as the 95% LoA was −23.96 to 23.36° and −1.15 to 0.37 mm, respectively.* Conclusion*. With the exception of astigmatism axis and WTW, anterior segment measurements taken by Sirius and IOLMaster devices showed good agreement and may be used interchangeably in patients with cataracts.

## 1. Introduction

Obtaining accurate and repeatable measurements of anterior segment parameters is a mandatory step in achieving the best outcomes in refractive anterior segment surgery. The corneal power (K) value is entered into any intraocular lens (IOL) power formula [1–4], while corneal astigmatism measurements are needed when planning toric IOLs implantation. Anterior chamber depth (ACD) is required by the Haigis [5], Holladay 2 [6], and Shammas formulae [7], which can be used to assess the risk for angle closure [8, 9] and investigate changes in the anterior eye segment during accommodation [10]. Corneal power is important to the evaluation of corneal ectasia and the diagnosis of keratoconus [11]. Finally, the white to white (WTW) distance can be used to estimate phakic intraocular lens (IOL) size [12].

Anterior segment measurements can now be obtained by a number of instruments, including manual and automated keratometers, corneal topographers, Scheimpflug cameras, and optical coherence tomographers. Measurement agreement among these devices is important in clinical practice. The IOLMaster (Carl Zeiss Meditec, Germany) and Sirius (CSO, Italy) are two validated and widely used instruments [13–16]. To our knowledge, their measurements have never been compared in a cataractous population. Therefore, the current study was performed to evaluate and compare K, axis of the corneal astigmatism, ACD, and WTW measurements of the Sirius and IOLMaster devices.

## 2. Patients and Methods

This prospective study adhered to the tenets of the Declaration of Helsinki and was approved by the Research Review Board at Wenzhou Medical University. After learning about the study procedure and purpose, all subjects provided written informed consent.

Ninety eyes of 90 patients (40 men and 50 women) with a mean age of 65.46 ± 9.78 years (range, 38 to 84 years) were included in this study. All subjects received a full ophthalmologic examination and all eyes involved in the study had clinically significant cataracts. The exclusion criteria included active inflammation, previous refractive surgery, corneal scarring, a history of contact lens wear within the previous 4 weeks, and severe systemic diseases that made patients intolerant to the operation. Moreover, those patients who could not successfully fixate their eyes or had severe blepharophimosis were also excluded.

## 3. Measurement Devices

The IOLMaster measures the radius of anterior corneal curvature and the respective axial orientation using the data from six light reflections oriented in an approximately 2.3 mm-diameter hexagonal pattern. The corneal refractive index used to calculate the keratometric power and keratometric astigmatism for this study was 1.3375. The device measures the ACD (i.e., distance from the corneal epithelium to the anterior lens surface) through a 0.7 mm-wide lateral slit illumination at 30°.

The Sirius combines a single-Scheimpflug rotating camera with Placido disk topography to measure and image the anterior eye segment. Within a single scan, it can simultaneously acquire more than 30,000 points on the corneal anterior and posterior surfaces and 25 radial sections of the cornea and anterior chamber. The system acquires the radius curvature measurements in the flat and steep meridians on a 3.0 mm-diameter field of the central cornea [17]. The corneal power and astigmatism were calculated using the 1.3375 keratometric refractive index.

## 4. Measurement Technique

Measurements were taken by the IOLMaster and Sirius in a random order. Each patient was guided to a seat in front of the equipment and placed their chin on a chinrest and their forehead against the forehead strap. The patients were instructed to fixate on an internal fixation target within each device and permitted to blink completely just before each measurement to spread an optically smooth tear film over the cornea and keep the eye open during image acquisition.

Measurements for both devices were performed according to their respective manufacturer's guidelines. Only high-quality measurements were included in the subsequent analysis. With Sirius, scans had to show the “OK” signal, meaning that Placido and Scheimpflug acquisition was above the required quality specification for coverage and centration. With IOLMaster, the optimum measurement setting (green traffic light) had been reached. To avoid the effects of diurnal variation in corneal shape and thickness, we completed the entire scanning procedure within 15 minutes in each case [18]. After the first device's measurements were taken, the patients were asked to rest with their eyes before undergoing an examination by the other one 3 minutes later. All measurements were performed by the same experienced examiner between 10 a.m. and 5 p.m. to eliminate operator-induced error and diurnal variations.

## 5. Statistical Analysis

Statistical analyses were performed using SPSS software (version 17; SPSS Inc., Chicago, IL, USA). *P* values < 0.05 were considered statistically significant. The normality of all data distributions was confirmed using the Kolmogorov-Smirnov test (*P* > 0.05).

Comparisons between the Sirius and IOLMaster measurements were conducted using paired *t*-tests to assess the mean differences in the anterior segment parameters and Bland-Altman plots to assess the degree of agreement between the two methods [19]. In this analysis, bias was defined as a significant difference in the means of the two methods; 95% limits of agreement (LoA) were calculated as the mean difference ±1.96 SD.

## 6. Results

Table 1 shows the measured parameters: mean ACD, flattest keratometry (Kf), steepest keratometry (Ks), mean keratometry (Km), astigmatism axis, and WTW.  Table 2 shows the mean difference, SD, *P* values, and 95% LoA. The ACD, Kf, Ks and Km taken with the Sirius were statistically significantly higher than that taken with the IOLMaster (*P* < 0.05); however, the Sirius significantly underestimated the WTW values compared with the IOLMaster biometer (*P* < 0.05).

**Figure 1:**
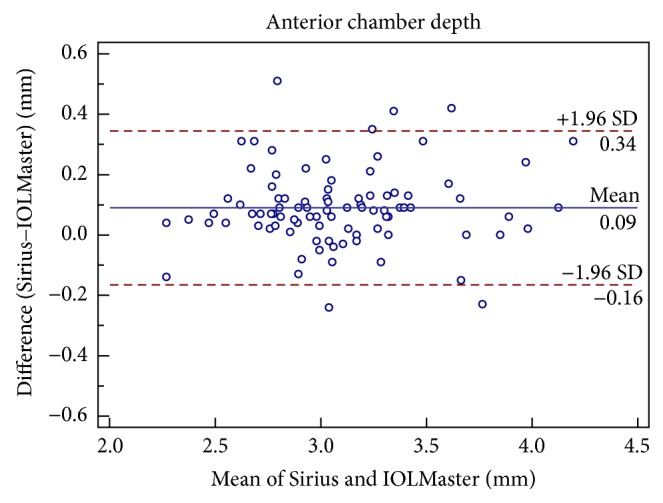
Bland-Altman plots of agreement of anterior chamber depth measurements taken with the Sirius Scheimpflug/Placido photography-based topography system and IOLMaster partial coherence interferometry. The solid line indicates the mean difference (bias). The upper and lower lines represent the 95% limits of agreement.

**Figure 2:**
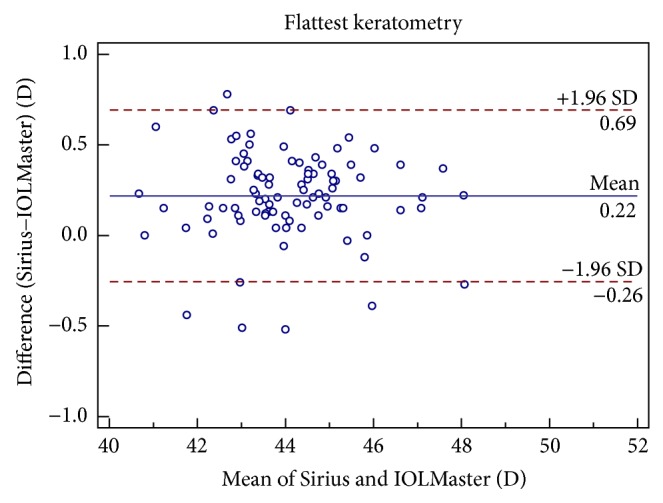
Bland-Altman plots of agreement of keratometry values along the flattest meridian taken with the Sirius Scheimpflug/Placido photography-based topography system and IOLMaster partial coherence interferometry. The solid line indicates the mean difference (bias). The upper and lower lines represent the 95% limits of agreement.

**Figure 3:**
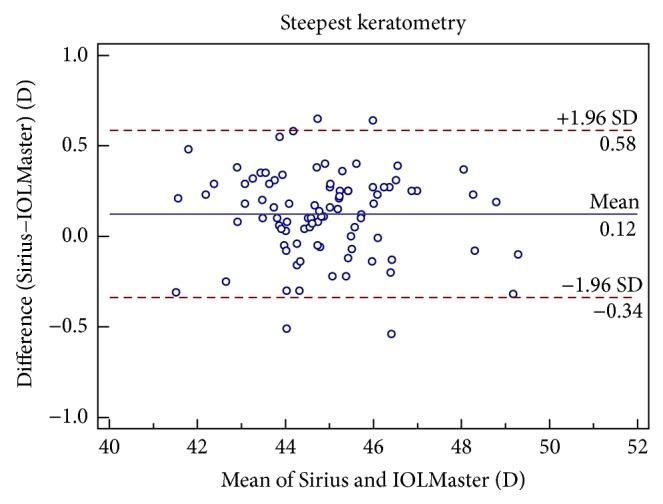
Bland-Altman plots of agreement of keratometry values along the steepest meridian taken with the Sirius Scheimpflug/Placido photography-based topography system and IOLMaster partial coherence interferometry. The solid line indicates the mean difference (bias). The upper and lower lines represent the 95% limits of agreement.

**Figure 4:**
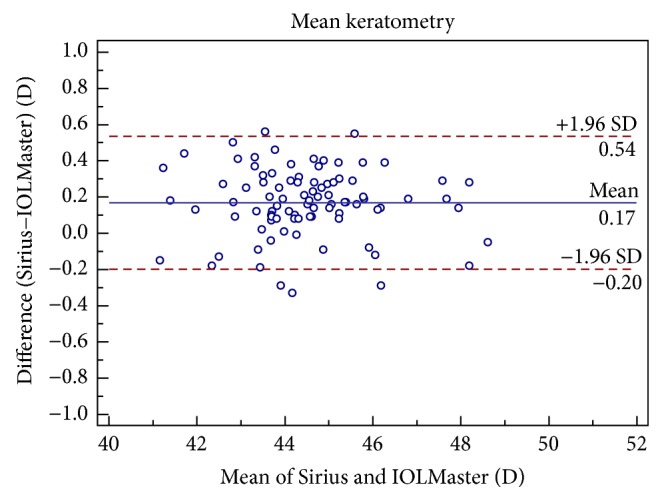
Bland-Altman plots of agreement of mean Keratometry readings taken with the Sirius Scheimpflug/Placido photography-based topography system and IOLMaster partial coherence interferometry. The solid line indicates the mean difference (bias). The upper and lower lines represent the 95% limits of agreement.

**Figure 5:**
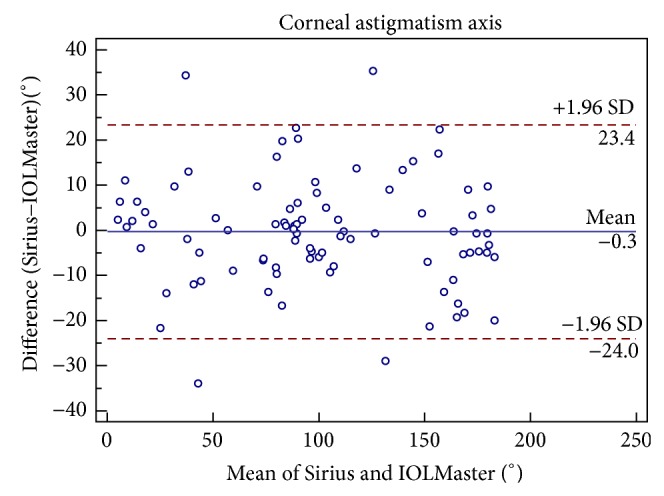
Bland-Altman plots of agreement of mean corneal astigmatism axis readings taken with the Sirius Scheimpflug/Placido photography-based topography system and IOLMaster partial coherence interferometry. The solid line indicates the mean difference (bias). The upper and lower lines represent the 95% limits of agreement.

**Figure 6:**
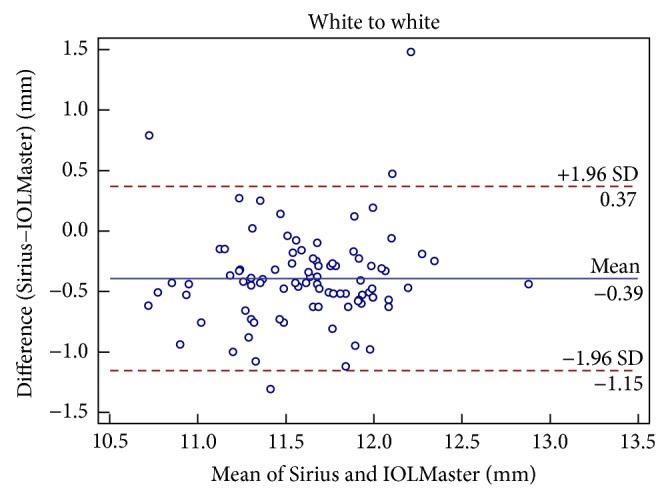
Bland-Altman plots of agreement of white to white readings taken with the Sirius Scheimpflug/Placido photography-based topography system and IOLMaster partial coherence interferometry. The solid line indicates the mean difference (bias). The upper and lower lines represent the 95% limits of agreement.

Figures 1, 2, 3, 4, 5, and 6 show Bland-Altman plots for the anterior parameter comparisons between the Sirius and IOLMaster. The magnitude of the 95% LoA was greatest for the astigmatism axis and WTW. Except for the astigmatism axis and WTW value, the Bland-Altman plots showed that the mean differences between the two devices were not significantly different for other comparisons of anterior parameters, which implied good agreement.

## 7. Discussion

This investigation showed that, with the exception of astigmatism axis and WTW, the anterior segment measurements taken by the Sirius are similar to those obtained by the IOLMaster, which means that they show good agreement and may be used interchangeably in patients with cataracts. Achieving similar measurements could be useful in several clinical applications, including intraocular lens calculation.

Accurate and precise determination of the anterior ocular segment is fundamental to many clinical and research applications in ophthalmology. The Sirius device is both noncontact and easy to use and showed good repeatability of the anterior segment measurements in healthy eyes [20] as well as those after refractive surgery [16] or with keratoconus [21]. As far as we are concerned, this is the first prospectively designed comparative study of the differences and agreement of the measurement of the anterior segment using both devices in patients with cataracts.

In the current study, the corneal power measurements (Ks, Km) obtained by the Sirius and IOLMaster showed a high level of agreement. Although such good agreement suggests that their measurements could be used interchangeably, we always recommend optimizing the IOL constants when shifting from one instrument to another. The K value obtained by the Sirius device was slightly higher than that produced by the IOLMaster, but the difference in averages was too small to be clinically relevant. These findings are consistent with previous studies of the IOLMaster [17, 22]. However, for corneal astigmatism axis, the range of the 95% LoA was too broad to be accepted in clinical practice [23].

Shirayama et al. [22] compared the corneal powers obtained using four different instruments in 20 healthy volunteers and found that the values obtained by the Galilei (Ziemer, Port, Switzerland), which uses dual Scheimpflug cameras and a Placido disk, were highly comparable with those obtained by the IOLMaster. The mean central corneal powers difference between them was only 0.12 diopter (D). This finding implied that the technique combining a Scheimpflug camera and a Placido disk could obtain valid and accurate corneal power in clinical application. In addition, Symes et al. [24] found that the Scheimpflug system was comparable to the IOLMaster device and might have better accuracy in eyes with higher delta K values (mean 2.13 diopters) and a greater degree of preoperative astigmatism. The values obtained from the Sirius device in our study were a bit lower than those of De la Parra-Colin et al. [20], which may be due to many reasons. That study compared the anterior segment biometry parameters obtained from the Sirius and a Pentacam (Oculus, Germany) in unoperated eyes of healthy subjects, while we recruited patients with cataracts. Moreover, the age range was also different, as that in the previous study was younger (mean age, 24.6 ± 1.64 years). Because a cross-sectional sampling showed that corneal curvature tended to increase with age, the discrepancy would be due to the differences in study populations [25].

With regard to ACD measurements, the mean values were 3.13 ± 0.41 mm versus 3.04 ± 0.40 mm by the Sirius and IOLMaster, respectively. It was a little bit higher when the ACD was measured with the Sirius but the difference was not statistically significant. We attributed this divergence to alignment differences, as the IOLMaster measures ACD along the visual axis [26] whereas the Sirius measures ACD along the optical axis, which usually represents the deepest central ACD.

The other variable, instrument-specific corrective factors, may be responsible for those systematic differences. Although no studies have directly compared the ACD measurement by the Sirius and IOLMaster, indirect study results did provide some indications. Utine et al. [27] compared ACD measurements taken with the Pentacam, IOLMaster, and Orbscan in 42 volunteers. Although the average IOLMaster ACD measurement was 0.11 mm smaller than that of the Pentacam, the observed mean error was too small to create any noticeable difference in refractive outcome in cataract surgery in the clinical setting. Interestingly, De la Parra-Colin et al. [20] evaluated the repeatability and comparability of ACD measured by the Sirius and Pentacam and found that both devices showed adequate agreement in unoperated healthy eyes. These findings implied that the Sirius combined with Scheimpflug/Placido was comparable with IOLMaster in ACD measurements.

Although recent studies have shown that the WTW cannot accurately predict the real sulcus-to-sulcus distance [28], it remains an important biometric parameter for phakic IOL diameter calculation [29]. Because patients are not comfortable due to direct contact measurements by ultrasound biomicroscopy [30], most surgeons rely on noncontact devices such as the IOLMaster. Baumeister et al. [31] compared manual and automated methods to measure the WTW and found that automated devices provide more precise and reliable results and that the IOLMaster has the highest reliability for measuring corneal diameter. In our study, we found that this measurement was 11.42 ± 0.46 mm using the Sirius, lower than the average value obtained by the IOLMaster (11.8 ± 10.42 mm). The Sirius could be expected to read as much as 0.37 mm above to 1.15 mm below the IOLMaster for WTW measurement. This disparity was statistically significant and may have clinical implications (e.g., relying on Sirius measurements may lead to an incorrect sizing of phakic IOLs). Thus, these devices should not be considered interchangeable for WTW assessments in clinical practice. Although we do not yet know the exact reason for the differences in the values obtained by these two imaging devices, we speculate that fundamental methods for acquiring and analyzing images were responsible to the disparity.

This study has some limitations. From a practical point of view, when the Sirius is used to calculate IOLs, the use of other devices to obtain the axial length is also required. As such, having the ability to measure the axial length would expand the clinical application of the Sirius. Studies have shown that the Scheimpflug photography feature provided precise and valid measurements for IOL calculation [32]. Savini et al. [33] enrolled 43 consecutive patients who were scheduled to undergo phacoemulsification to evaluate the accuracy of the Galilei dual Scheimpflug analyzer and a Placido disk corneal topography system (Optikon 2000 SpA; Keratron, Italy) for IOL calculation, which found that corneal power measurements provided by the Scheimpflug camera and Placido disk corneal topographer obtained accurate IOL power calculation. The same team also confirmed that corneal power measurements provided by the Sirius were successfully entered into third-generation IOL power calculation formulas in eyes with cataracts. Moreover, optimization of constants is still required for calculating IOLs. Different constants must be used for adjusting the postoperative IOL position. On the other hand, some studies suggested that the IOLs could influence measurement accuracy [34, 35]. Since both devices were not used in the patients after cataract surgery in the same prospective study, we could not determine which one was better for predicting the IOL power calculation in patients with cataracts, which requires further investigation.

In summary, with the exception of astigmatism axis and WTW measurements, the Sirius Scheimpflug-Placido topographer and IOLMaster partial coherence interferometer showed good agreement in anterior segment measurements, indicating that they may be used interchangeably in most clinical applications.

## Figures and Tables

**Table 1:** Descriptive statistics of ocular measurements by Sirius Scheimpflug/Placido photography-based topography system and IOLMaster partial coherence interferometry.

Parameter	Mean ± SD	Minimum	Maximum
Sirius			
ACD (mm)	3.13 ± 0.41	2.20	4.35
Kf (D)	44.19 ± 1.50	40.79	48.16
Ks (D)	44.95 ± 1.55	41.36	49.24
Km (D)	44.57 ± 1.50	41.09	48.59
Axis (°)	99.85 ± 52.50	3.7	180.0
WTW (mm)	11.42 ± 0.46	10.41	12.95
IOLMaster			
ACD (mm)	3.04 ± 0.40	2.25	4.08
Kf (D)	43.97 ± 1.51	40.56	48.21
Ks (D)	44.83 ± 1.57	41.46	49.34
Km (D)	44.40 ± 1.51	41.06	48.64
Axis (°)	100.16 ± 53.46	3.7	180.0
WTW (mm)	11.81 ± 0.42	10.33	13.10
AL (mm)	23.47 ± 1.11	21.28	28.36

ACD = anterior chamber depth, Kf = flattest keratometry, Ks = steepest keratometry, Km = mean keratometry, WTW = white to white, AL = axial length, and SD = standard deviation.

**Table 2:** The mean difference, limits of agreement (LoA), paired *t*-test for these differences, and their significance for difference parameters between Sirius Scheimpflug/Placido photography-based topography system and IOLMaster partial coherence interferometry.

Device pairings	Mean difference ± SD	*P* value	95% LoA
ACD (mm)	0.09 ± 0.13	<0.05	−0.16 to 0.34
Kf (D)	0.22 ± 0.24	<0.05	−0.26 to 0.69
Ks (D)	0.12 ± 0.24	<0.05	−0.34 to 0.58
Km (D)	0.17 ± 0.19	<0.05	−0.20 to 0.54
Axis (°)	−0.30 ± 12.07	0.813	−23.96 to 23.36
WTW (mm)	−0.39 ± 0.39	<0.05	−1.15 to 0.37

ACD = anterior chamber depth, Kf = flattest keratometry, Ks = steepest keratometry, Km = mean keratometry, WTW = white to white, and SD = standard deviation.
